# Analysis of Risk Factors of Peripherally Inserted Central Catheter Induced Catheter-related Infection in Patients with Leukemia

**Published:** 2017-04

**Authors:** Lanlan ZHOU, Mingjun WANG, Aping LI

**Affiliations:** 1. Dept. of Hematology and Oncology, The Second People’s Hospital of Hefei, Hefei, Anhui, China; 2. Dept. of Hematology, Liaocheng People’s Hospital, Liaocheng, Shandong, China; 3. Dept. of Neurosurgery, People’s Hospital of Haiyang City, Haiyang, Shandong, China

**Keywords:** Leukemia, Peripherally inserted central catheter (PICC), Catheter-related infection (CRI), Nursing, Multivariate logistic regression

## Abstract

**Background:**

Peripherally inserted central catheter (PICC) is commonly used in nursing for patients with leukemia. The purpose of this study was to investigate the risk factors of Catheter-related infection (CRI) in patients with leukemia and to provide some nursing strategies based on the results.

**Methods:**

Clinical data from 140 patients with leukemia between May 2014 and July 2016 in Haiyang People’s Hospital, China were retrospectively analyzed. We employed univariate analysis to explore the relationship of various factors, including leukemia types, puncture times, underlying diseases, Catheter indwelling time, hormones use, chemotherapy use, immune functions and seasons, with the incidence of CRI. Further, multivariate logistic regression analysis was conducted to identify the potential independent risk factors of CRI. Bacterial culture was performed for etiological detection.

**Results:**

Among the 140 patients with leukemia, 25 cases were diagnosed as CRI, with the incidence of 17.9%. Univariate analysis showed that puncture times, underlying diseases, catheter indwelling time, hormones use, chemotherapy use, immune functions and seasons were significantly correlated with the incidence of CRI. Multivariate logistic regression analysis revealed that immune functions, puncture times and seasons were independent risk factors for CRI. Etiological bacterial culture detected 20 strains of bacteria (*Staphylococcus aureus*: n=10, *Klebsiella pneumonia*: n=4, *Coryne-bacterium*: n=2 and other species: n=4) in 25 cases diagnosed with CRI.

**Conclusion:**

Based on risk factors of CRI and its etiological distribution, appropriate nursing measures can be taken to reduce the incidence of CRI in patients with leukemia.

## Introduction

Leukemia is a heterogeneous group of hematopoietic malignancies that include many diverse and biologically distinct subgroups, including acute lymphoblastic leukemia (ALL), chronic lymphoblastic leukemia (CLL), acute myeloid leukemia (AML), and chronic myeloid leukemia (CML) (1). For different types and different ages, the incidence of leukemia ranges. American Cancer Society (ACS) showed that nearly 30,800 individuals per year were diagnosed with leukemia in the United States. Among, 21,700 of them are deadly cases (2). With the use of risk-directed treatment and developed supportive care, treatment outcomes for leukemia have improved substantially. During the past decade, epidemiological analysis has revealed a significant increase of the 5-year-survival rates (3). Especially for children with ALL receiving protocol-based therapy, the 5-year-survival rate has been increased to nearly 80% (4).

Leukemia patients often need to be infused with a large number of strongly stimulating chemotherapeutic agents during the course of treatment, while the transfusion extravasation, repeated venepuncture and other factors in the chemotherapy process provoke the occurrences of phlebitis and other complications easily, which cause a strong impact on the therapeutic effect and prognosis (5–7). Although peripherally inserted central catheter (PICC) brings a lot of convenience for patients and clinical nursing, there are still some corresponding complications, such as long-term indwelling can cause mechanical or thrombophlebitis, catheter misplacement, fall-off of catheter, catheter blockage and so forth, if handled improperly, it will bring physical and mental suffering and economic losses to patients, and may even lead to disputes between doctors and patients (8–11).

Leukemia patients often require intravenous infusion of a large number of chemotherapy drugs with stronger irritation. Given that the patients with low platelet count, decreased granulocyte, low autoimmune function and high blood viscosity often need to be treated with intravenous infusion of high concentration of nutrient liquid and blood products, the complications after in-dwelling catheter, such as puncture point bleeding, catheter-related infection, catheter blockage, non-infective phlebitis and others often occur (5,11). Therein, catheter-related infection (CRI) is one of the common complications of long time indwelling operation of PICC. The incidence of PICC catheter-related infection of leukemia is higher than that of other diseases (12).

This study aimed to investigate the risk factors of PICC catheter-related infection of patients with leukemia, and to put forward some nursing strategies in accordance with the related research results.

## Methods

### Patients

We retrospectively collected clinical data from 140 patients with leukemia between May 2014 and July 2016.Among there were 89 males and 51 females, aged 14–86 yr (58.91±12.09). Other information, including leukemia types, puncture times, underlying diseases, Catheter indwelling time, hormones use, chemotherapy use, immune functions and seasons, were also recorded (Table 1).

**Table 1:** Clinical data of patients with leukemia (%)

**Clinical data**		**Case (n)**	**Constituent ratios (%)**
Types	ALL	67	47.9
	CLL	11	7.9
	AML	44	31.4
	CML	18	12.8
Gender	Male	89	63.6
	Female	51	36.4
Age (yr)	>50	73	52.1
	≤50	67	47.9
Punctural times	1	108	77.1
	≥1	32	22.9
Underlying diseases	Yes	33	23.6
	No	107	76.4
Catheter indwelling time (Month)	<1	42	30.0
	≥2	98	70.0
History of use of hormones	Yes	61	43.6
	No	79	56.4
Use of chemotherapy drugs	Yes	121	86.4
	No	19	13.6
Lower immune function	Yes	117	83.6
	No	23	16.4

This study was approved by the Ethics Committee of Haiyang People’s Hospital. Signed written informed consents were obtained from all participants before the study.

### Samples collection and bacterial culture

For those confirmed cases with CRI, purulent secretion nearby the puncture position was collected for bacterial culture etiologic examination. CRI diagnosis criteria were according to a previous report: Purulent secretion and diffuse erythema appeared nearby the puncture site, accompanied by pain, tenderness, elevated body temperature and positive bacterial culture results.

### Statistical analysis

Quantitative Data were expressed as mean ± standard deviation. Percentage (%) was used to express the enumeration data and chi-square test was used for data analysis. Univariate analysis or multi-factor logistic regression analysis was conducted using SPSS software (Version 19.0IBM, Armonk, NY, USA). *P* values < 0.05 were considered statistically significant.

## Results

### Total infection rate of CRI

Among a total of 140 patients with leukemia, 25 cases were diagnosed as CRI. The total incidence was 17.9%. The incidence for subgroups, such as different leukemia types, genders and ages, were detailed shown in Table 2.

**Table 2:** Univariate analysis of the related factors of COI in the patients with leukemia and the infection rates (%)

**Relative factors**		**Cases (n)**	**Infection number**	**Infection rate**	**χ**^**2**^ **value**	***P* value**
Types	ALL	67	12	14.9	3.04	>0.05
	CLL	11	2	18.2		
	AML	44	8	18.1		
	CML	18	3	16.7		
Gender	male	89	16	17.98	1.23	>0.05
	Female	51	9	17.65		
Ages (Year)	>50	73	14	19.18	2.09	>0.05
	≤50	67	11	16.41		
Puncture times(N)	1	108	16	14.81	6.07	<0.05
	≥1	32	9	28.13		
Underlying diseases	Yes	33	10	30.30	5.44	<0.05
	No	101	15	14.85		
Catheter indwelling time (Mont)	≥2	98	21	21.43	5.31	<0.05
	<2	42	4	9.52		
History of use of hormones	Yes	61	19	31.15	5.99	<0.05
	No	79	6	7.59		
Use of chemotherapy drugs	Yes	121	23	19.01	3.89	<0.05
	No	19	2	10.53		
Immune function	Normal	23	1	4.35	6.58	<0.05
	lower	117	24	20.51		
Seasons	Spring	30	5	16.67	7.18	<0.05
	Summer	27	14	51.85		
	Autumn	50	4	8.00		
	Winter	43	2	4.65		

### Univariate analysis

Univariate analysis results showed that puncture times, factors of underlying diseases, catheter indwelling time, hormones use, chemotherapy use, immune functions and seasons were all significantly correlated with the incidence of CRI (*P*<0.05).

For those who received puncture for over 2 times, combined with underlying diseases, used hormones, treated with chemotherapy, and cases with impaired immune functions and received PICC in summer, the incidence of CRI significantly increased. Detailed information was shown in Table 2.

### Multivariate logistic regression analysis

To further identify independent risk factors of CRI for leukemia patients, we conducted multivariate logistic regression analysis. Results indicated that immune functions, puncture times and seasons were independent risk factors of CRI (Table 3).

**Table 3:** Multivariate logistic regression analysis of the clinical factors for the PICC-related infections in the patients with leukemia

**Clinical factors**	**β**	**s x̄**	**OR**	**95% CI**	**Χ**^**2**^	***p* value**
Puncture time	0.512	0.090	1.533	1.274∼1.785	6.72	0.090
Seasons	0.616	0.035	1.636	1.154∼1.573	7.09	0.008
Immune function	0.549	0.043	1.584	1.657∼1.918	6.88	0.009

### Etiological analysis of CRI

We next collected samples of purulent secretion from puncture sites to explore the etiological distribution. Bacterial culture obtained 20 strains of bacteria in 25 cases diagnosed with CRI. Among, there were *10 Staphylococcus aureus*, 4 *Klebsiella* pneumonia, 2 *Corynebacterium* and 4 other species (Table 4).

**Table 4:** Constituent ratio of the pathogens causing the PICC-related infections (%)

**Pathogens**	**Strains number**	**Constituent ratio (%)**
*Staphylococcus aureus*	10	50.00
*Klebsiella pneumonia*	4	20.00
*Corynebacterium*	2	10.00
Other species	4	20.00
Total	20	100.00

## Discussion

Catheter-related infection is a common complication of long-term indwelling catheter of PICC, and for some severe cases, it can lead to hematosepsis or systemic infectious disease (8, 11). Therefore, to explore the main influencing factors of catheter-related infection and put forward some reasonable nursing strategies according to the risk factors of infection has an important clinical significance in controlling and preventing the occurrence of infection.

Reasonable nursing strategy is of great significance in the prevention and control of PICC catheter-related infection (13). According to the influencing factors of the catheter-related infection, the nursing strategies are proposed as follows (14–16):

- Because of the lack of autoimmune function of patients with hematologic neoplasm, the pathogen is easy to attack, which will cause the increase of the occurrence risk of hospital infectious disease and its complications as well as the clinical adverse events; meanwhile, under the influence of adverse drug reactions, the phenomenon of bone marrow suppression often occurs, which will cause the gradual increase of infection rate. Therefore, during the period of chemotherapy treatment for patients, we need to monitor the hematocyte count strictly, give patients with appropriate symptomatic and supportive treatment, monitor the changes of vital signs of patients strictly, observe whether the puncture site bleeding occurs and accompanied by symptoms as red, swell, hot and pain, according to doctor’s advice to give the immune enhancer, give the food which is easy to be digested and has high nutrition, thereby improving the immune function of patients.
- In summer, the risk of catheter-related infection and other hospital infections is significantly increased. The significant increase of growth and reproduction of pathogenic microorganisms caused by hot weather will easily cause the occurrence of skin sweating and skin adverse reactions, causing the fall-off of catheter, and then bacteria are more likely to invade the mouth of the catheter. Therefore, to keep the ventilation of ward, increase the frequency of changing dressing in hot summer or at the time of sweating, and perform the local changing dressing using chlorotetracycline ointment or bactroban ointment are conducive to decrease the occurrence of bacterial infection.
- The non-standard operation of changing dressing, the incomplete or non-standard disinfection of hands before the operation of puncture and indwelling catheter, the non-standard aseptic disinfection and other factors increase the incidence of PICC catheter-related infection.

## Conclusion

We need to perform the aseptic operation strictly, keep the clean sticking, strengthen the propaganda and education of health knowledge, pay attention to the prevention and control of catheter-related infection strictly, maintain the cleanliness of the surrounding environment or personal hygiene, reduce PICC catheter indwelling time and avoid the use of hormone drugs.

## Ethical considerations

Ethical issues (Including plagiarism, informed consent, misconduct, data fabrication and/or falsification, double publication and/or submission, redundancy, etc.) have been completely observed by the authors.
